# P-670. Differences in Adenovirus Type Detection in Children with Acute Respiratory Illness versus Healthy Controls: New Vaccine Surveillance Network (NVSN) 2016-2019

**DOI:** 10.1093/ofid/ofae631.866

**Published:** 2025-01-29

**Authors:** Adam Gailani, Tess Stopczynski, Varvara Probst, Justin Z Amarin, Laura S Stewart, Rangaraj Selvarangan, Jennifer E Schuster, Christopher J Harrison, Marian G Michaels, John V Williams, Pedro A Piedra, Leila C Sahni, Mary A Staat, Elizabeth P Schlaudecker, Geoffrey A Weinberg, Peter G Szilagyi, Janet A Englund, Eileen J Klein, Ariana Perez, Benjamin R Clopper, Heidi L Moline, James Chappell, Andrew J Spieker, Natasha B Halasa

**Affiliations:** Vanderbilt University Medical Center, Nashville, Tennessee; Vanderbilt University Medical Center, Nashville, Tennessee; Nationwide Children's Hospital, Columbus, Ohio; Vanderbilt University Medical Center, Nashville, Tennessee; Vanderbilt University Medical Center, Nashville, Tennessee; Children’s Mercy Kansas City, Kansas City, Missouri; Children’s Mercy Kansas City, Kansas City, Missouri; Children's Mercy Hospital, Kansas City, Missouri; UPMC Children's Hospital of Pittsburgh, Pittsburgh, Pennsylvania; University of Pittsburgh, Pittsburgh, Pennsylvania; Baylor College of Medicine, Houston, Texas; Baylor College of Medicine and Texas Children’s Hospital, Houston, Texas; Cincinnati Children’s Hospital Medical Center, Cincinnati, Ohio; Cincinnati Children's Hospital Medical Center, Cincinnati, Ohio; University of Rochester School of Medicine & Dentistry, Rochester, NY; UCLA School of Medicine, Agoura Hills, California; Seattle Children’s Hospital, Seattle, Washington; University of Washington School of Medicine, Seattle, Washington; CDC, Avondale Estates, Georgia; US Centers for Disease Control & Prevention, Buffalo, New York; Centers for Disease Control and Prevention, Atlanta, Georgia; Vanderbilt University Medical Center, Nashville, Tennessee; Vanderbilt University Medical Center, Nashville, Tennessee; Vanderbilt University Medical Center, Nashville, Tennessee

## Abstract

**Background:**

Human adenovirus (HAdV) is a common cause of acute respiratory illness (ARI) in children, ranging from asymptomatic to life-threatening disease. While previous studies have examined asymptomatic vs HAdV ARI infections, few have analyzed HAdV species and type. We compared frequencies of common HAdV ARI types among children with ARI vs. healthy controls (HCs).

**Figure:**
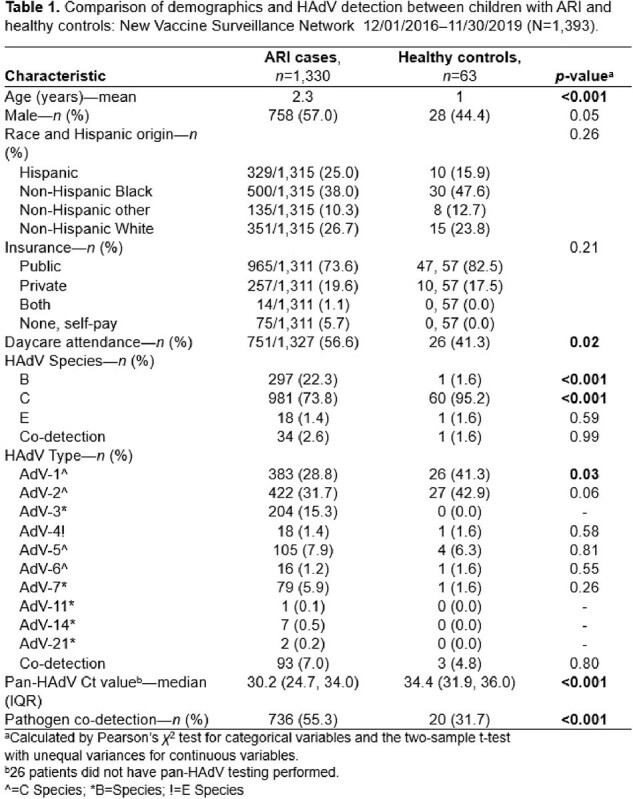

Comparison of demographics and HAdV detection between children with ARI and healthy controls: New Vaccine Surveillance Network 12/01/2016–11/30/2019 (N=1,393).

**Methods:**

Data were analyzed from the New Vaccine Surveillance Network, a multicenter, prospective surveillance network at 7 U.S. children’s hospitals that enrolled children < 18 years with ARI (fever and/or respiratory symptoms) from emergency departments or inpatient settings during 12/01/16–11/30/19. HCs (ARI symptom free ≥3 days and no vomiting/diarrhea ≥14 days) were matched by age and near date of enrollment, and enrolled from outpatient clinics. Nasal and/or throat swabs were tested using molecular assays for HAdV and other respiratory viruses. HAdV-positive specimens were tested by real-time PCR for 11 common respiratory HAdV types representing species B, C, and E based on unique sequences in the hexon gene. Demographics, HAdV species, and HAdV types from HAdV-positive children with ARI were compared to those from HAdV-positive HCs.

**Figure:**
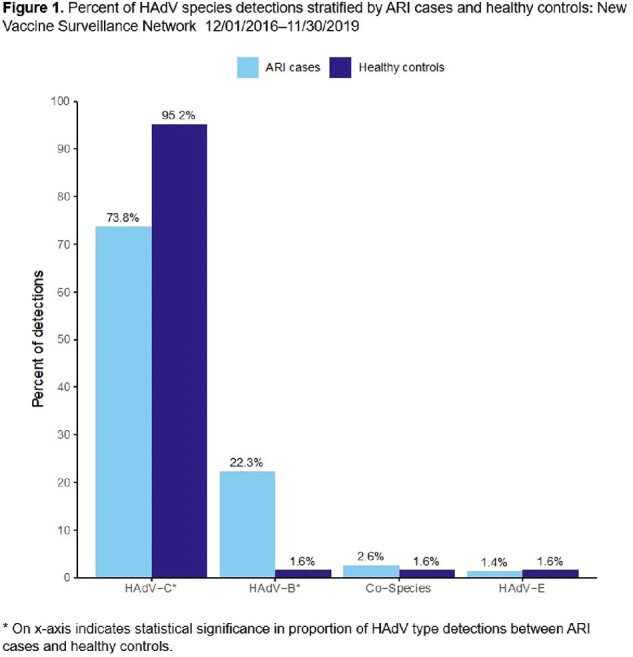

Percent of HAdV species detections stratified by ARI cases and healthy controls: New Vaccine Surveillance Network 12/01/2016–11/30/2019

**Results:**

1,330 HAdV detections from ARI cases and 63 from HCs were included. Children with HAdV ARI were older, more frequently attended daycare, had higher viral load (based on cycle threshold value), and had more frequent viral co-detection compared to HCs (**Table 1**). HAdV-C predominated in both ARI and HC groups (**Fig. 1**); HAdV-C2 was most common, followed by HAdV-C1 (**Fig. 2**). Among HAdV-positive patients, those with ARI more frequently had HAdV-B detection than HCs (22.3% vs. 1.6%, p< 0.001). HAdV-positive HCs more frequently had HAdV-C detected than HAdV-positive ARI cases (95.2% vs 73.8%, p< 0.001). HAdV-C1 was more likely to be detected in HCs than those with ARI (28.9% vs. 41.3%, p=0.03), and HAdV-B3 was only detected in those with ARI.

**Figure:**
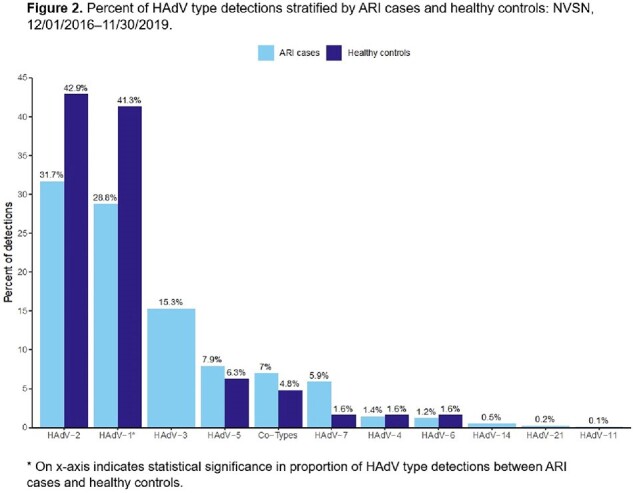

Percent of HAdV type detections stratified by ARI cases and healthy controls: NVSN, 12/01/2016–11/30/2019.

**Conclusion:**

While HAdV-C was most frequent in all children, HAdV-B was more often detected in those with ARI than HCs, suggesting that different HAdV species and types have different symptom phenotypes, including HAdV-B3. Further investigations into the identification of specific HAdV types responsible for ARI are necessary.

**Disclosures:**

**Rangaraj Selvarangan, BVSc, PhD, D(ABMM), FIDSA, FAAM**, Abbott: Grant/Research Support|Abbott: Honoraria|BioMerieux: Grant/Research Support|Cepheid: Grant/Research Support|Diasorin: Grant/Research Support|GSK: Advisor/Consultant|Hologic: Grant/Research Support|Luminex: Grant/Research Support|Qiagen: Grant/Research Support **Christopher J. Harrison, MD**, GSK: Grant/Research Support|Medscape: Honoraria|Merck: Grant/Research Support|Pfizer: Grant/Research Support|UpToDate: Honoraria **Pedro A. Piedra, MD**, Merck: Grant/Research Support|Novavax: Grant/Research Support|Sanofi-Pasteur: Grant/Research Support|Shionogi: Grant/Research Support **Mary A. Staat, MD, MPH**, Cepheid: Grant/Research Support|Merck: Grant/Research Support|Pfizer: Grant/Research Support|Up-To-Date: Honoraria **Elizabeth P. Schlaudecker, MD, MPH**, Pfizer: Grant/Research Support|Sanofi Pasteur: Advisor/Consultant **Geoffrey A. Weinberg, MD**, Inhalon: Advisor/Consultant|Merck & Company: Honoraria for textbook chapter preparation **Janet A. Englund, MD**, Abbvie: Advisor/Consultant|AstraZeneca: Advisor/Consultant|AstraZeneca: Grant/Research Support|GlaxoSmithKline: Advisor/Consultant|GlaxoSmithKline: Grant/Research Support|Meissa Vaccines: Advisor/Consultant|Merck: Advisor/Consultant|Pfizer: Board Member|Pfizer: Grant/Research Support|Pfizer: Speaker at meeting|SanofiPasteur: Advisor/Consultant|Shinogi: Advisor/Consultant **James Chappell, MD, PhD**, Merck: Grant/Research Support **Natasha B. Halasa, MD, MPH**, Merck: Grant/Research Support

